# The *LIKE SEX FOUR2* regulates root development by modulating reactive oxygen species homeostasis in *Arabidopsis*

**DOI:** 10.1038/srep28683

**Published:** 2016-06-28

**Authors:** Pingzhi Zhao, Lubomir N. Sokolov, Jian Ye, Cheng-Yi Tang, Jisen Shi, Yan Zhen, Wenzhi Lan, Zhi Hong, Jinliang Qi, Gui-Hua Lu, Girdhar K. Pandey, Yong-Hua Yang

**Affiliations:** 1 NJU–NJFU Joint Institute for Plant Molecular Biology, State Key Laboratory of Pharmaceutical Biotechnology, School of Life Sciences, Nanjing University, Nanjing 210023, China; 2 State Key Laboratory of Plant Genomics, Institute of Microbiology, Chinese Academy of Sciences, Beijing 100101, China; 3 NJU–NJFU Joint Institute for Plant Molecular Biology, MOE Key Laboratory of Forest Genetics and Biotechnology, Nanjing Forestry University, Nanjing 210037, China; 4 Department of Plant Molecular Biology, University of Delhi South Campus, New Delhi 110021, India

## Abstract

Maintaining reactive oxygen species (ROS) homeostasis plays a central role in plants, and is also critical for plant root development. Threshold levels of ROS act as signals for elongation and differentiation of root cells. The protein phosphatase LIKE SEX FOUR2 (LSF2) has been reported to regulate starch metabolism in *Arabidopsis*, but little is known about the mechanism how LSF2 affect ROS homeostasis. Here, we identified that LSF2 function as a component modulating ROS homeostasis in response to oxidative stress and, thus regulate root development. Compared with wild type *Arabidopsis*, *lsf2-1* mutant exhibited reduced rates of superoxide generation and higher levels of hydrogen peroxide upon oxidative stress treatments. The activities of several antioxidant enzymes, including superoxide dismutase, catalase, and ascorbate peroxidase, were also affected in *lsf2-1* mutant under these oxidative stress conditions. Consequently, *lsf2-1* mutant exhibited the reduced root growth but less inhibition of root hair formation compared to wild type *Arabidopsis* plants. Importantly, protein phosphatase LSF2 interacted with mitogen-activated protein kinase 8 (MPK8), a known component of ROS homeostasis pathways in the cytoplasm. These findings indicated the novel function of LSF2 that controls ROS homeostasis to regulate root development.

Plants absorb water and nutrients from the soil through their roots and root hairs, which sense abiotic and biotic stress during plant life cycle. Reactive oxygen species (ROS) play critical roles in root growth and root hair development in plants^1^. The most common ROS are hydroxyl radical (·OH), superoxide radicals (O_2_^•−^), and hydrogen peroxide (H_2_O_2_). Compared with other types of ROS, H_2_O_2_ is more stable, but O_2_^•−^ is spontaneously or enzymatically dismutated to H_2_O_2_. ·OH in plants is directly responsible for cell wall loosening and cell expansion in the root elongation zone^2^. In *Arabidopsis thaliana* roots, O_2_^•−^ localizes predominantly to the apoplast of cell elongation zone, and H_2_O_2_ is present mainly in the differentiation zone and cell walls of root hairs^3^. Accumulation of H_2_O_2_ inhibits root growth but promotes the growth and formation of adventitious roots^4^. Studies in which ROS levels were modified by various treatments have demonstrated that O_2_^•−^ and H_2_O_2_ are involved in root growth and root hair development.

Low levels of ROS serve as regulatory signals for the cell, but high levels of ROS, representing byproducts of plant aerobic metabolism produced in response to environmental stresses, are undesirable or toxic^5^,^6^. When *Arabidopsis* leaves were exposed to the herbicide paraquat (methyl viologen, MV), H_2_O_2_ accumulation increased^7^. Salicylic acid (SA) is an oxidative signal inducer that is required for the development of systemic acquired resistance (SAR) against various pathogens through its interaction with NPR3 and NPR4^8^. Plants contain several ROS scavenging enzymes, including superoxide dismutase (SOD), ascorbate peroxidase (APX), and catalase (CAT). The SOD family of metalloenzymes, including iron SOD (Fe-SOD), manganese SOD (Mn-SOD), and copper-zinc SOD (Cu/Zn-SOD), specifically catalyze the dismutation of O_2_^•−^ to H_2_O_2_ and molecular oxygen (O_2_)^9^. APX is a scavenging peroxidase of H_2_O_2_ that uses ascorbate as a reductant and has a high affinity for H_2_O_2_. CAT is the key enzyme that scavenges H_2_O_2_, with a high reaction rate but low affinity for H_2_O_2_^10^. ROS act as either damaging or signaling molecules in plants depending on their levels, which in turn depend on the rates of production and scavenging of ROS.

The precise balance of ROS plays an important role in regulating plant growth and development. Recent studies suggest that ROS homeostasis regulates the transition from cell proliferation to cell differentiation in *Arabidopsis* roots. *Arabidopsis* UPBEAT1 (UPB1) controls the balance between O_2_^•−^ and H_2_O_2_ by repressing peroxidases; *upb1* mutant plants contain higher O_2_^•−^ levels and lower H_2_O_2_ levels in the root tip compared with the wild type plants, suggesting that ROS homeostasis is altered in these plants, leading to changes in root growth and development^11^. Lack of *RHD2/AtrbohC* in the *Arabidopsis rhd2* mutant causes it to have reduced ROS levels and atypical tubulin formation, affecting cell division of the root meristem^12^. PHYTOCHROME AND FLOWERING TIME1 (PFT1), which primarily controls the expression of class III peroxidase genes (such as *UPBEAT*) in *Arabidopsis*, regulates the expression of genes encoding redox-active proteins^13^. In the *pft1* mutant, the balance between O_2_^•−^ and H_2_O_2_ is altered, resulting in defective root hair differentiation. Over-expressing of *PLANT FERREDOXIN-LIKE PROTEIN* (*PFLP*) in *Arabidopsis* increases ROS production and significantly promotes root hair growth^14^. Taken together, these studies support the important role of ROS homeostasis in regulating root growth and development.

In *Arabidopsis*, protein phosphatases play key roles in regulating cellular activities and often affect ROS homeostasis, especially in response to environmental stimuli^15^,^16^,^17^. There are two known glucan phosphatases in plants: STARCH EXCESS4 (SEX4) and LIKE SEX FOUR2 (LSF2). SEX4 is a major phosphatase that binds to starch granules, and is regulated by redox^18^. It is a disulfide bridge in the SEX4 phosphatase domain that acts as a redox-dependent switch to regulate SEX4 activity^19^. LSF2, which is located in the chloroplast and cytoplasm, specifically hydrolyzes phosphate groups from the C3-position and is involved in transitory starch metabolism^20^. However, no study has proved that LSF2 activity is controlled by redox in plants^21^. In this present study, for the first time, we identify that LSF2 plays a role in ROS homeostasis to regulate root growth and root hair development in *Arabidopsis*.

## Results

### ROS status is balanced under exogenous oxidative stresses in *lsf2–1* mutant

**Figure 1:**
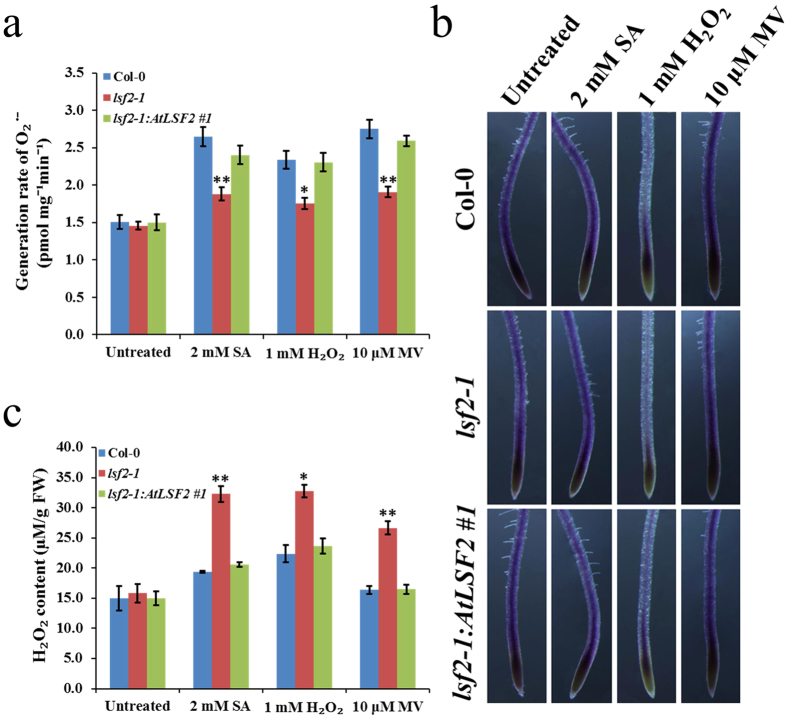
Generation rate of O_2_^•−^ and H_2_O_2_ content in plants. The generation rate of O_2_^•−^ (**a**) or endogenous H_2_O_2_ content (**c**) was examined after pre-treated with SA, H_2_O_2_ or MV (mean values ± SE, n = 3; student’s t-test, *P < 0.05; **P < 0.01). (**b**) NBT stained main roots that were pre-treated with SA, H_2_O_2_ or MV.

In order to determine the role of LSF2 in maintenance of ROS levels, we have isolated a homozygous T-DNA inserted mutant *lsf2-1* allele (SAIL line 595_F04 of *AT3G10940* from ABRC; Supplementary Fig. S1a–c) and four independent homozygous *lsf2-1:AtLSF2* complementary transgenic lines (Supplementary Fig. S2a,b). As it is already known that O_2_^•−^ is rapidly converted to H_2_O_2_ spontaneously or via dismutation by SOD enzymes. We used XTT as a probe to photometrically measure the generation rate of O_2_^•−^, as XTT is reduced to soluble formazan by O_2_^•−^. Under normal growth conditions, there were no statistical significant differences in O_2_^•−^ generation between *lsf2-1* and Col-0 (Fig. 1a, untreated). When treated with oxidative stress upon exposure to H_2_O_2_, the generation rate of O_2_^•−^ was obviously lower in *lsf2-1* than in Col-0 plants, and these differences were more significant after treatment with exogenous SA or MV (Fig. 1a). We also used NBT as a histochemical probe for O_2_^•−^, as it forms an insoluble blue/violet formazan product in the presence of O_2_^•−^. Strong blue/violet staining (revealing O_2_^•−^) was detected in the elongation zone under abiotic stress treatment, which also extended to the meristematic zone and differentiation zone (Fig. 1b). However, no significant differences were observed between the staining patterns of Col-0, *lsf2-1*, and *lsf2-1:AtLSF2* #1 roots. These results suggest that null mutation of *LSF2* in *Arabidopsis* results in more suppressed generation of O_2_^•−^ in response to exogenous oxidative stresses in leaves.

The accumulation of endogenous H_2_O_2_ in plants is an early response to environmental stimuli, such as pathogen attack and exogenous application of SA or MV^22^. We measured the contents of endogenous H_2_O_2_ using a peroxidase enzyme assay. No significant differences in H_2_O_2_ levels were detected between *lsf2-1* and Col-0 plants under normal growth conditions (Fig. 1c, untreated). However, compared with Col-0 plants, *lsf2-1* mutant plants produced higher levels of H_2_O_2_ under oxidative stress conditions (Fig. 1c). These results indicate that endogenous H_2_O_2_ accumulates much more rapidly in *lsf2-1* than in Col-0 plants under oxidative stress conditions, suggesting that LSF2 is involved in regulating endogenous H_2_O_2_ levels exposed to environmental stress. Analysis of *LSF2* expression patterns under these exogenous oxidative stresses (SA, H_2_O_2_ and MV) in Col-0 plants, the transcriptional expression of *LSF2* was strongly induced in leaves but slightly down-regulated in roots (Supplementary Fig. S1d). Therefore, we hypothesized that LSF2 might regulate ROS homeostasis (to a certain extent) by maintaining the balance between O_2_^•−^ levels and H_2_O_2_ levels in *Arabidopsis* leaves.

### Oxidative stress affects antioxidant enzyme activities in *lsf2-1* mutant

**Figure 2:**
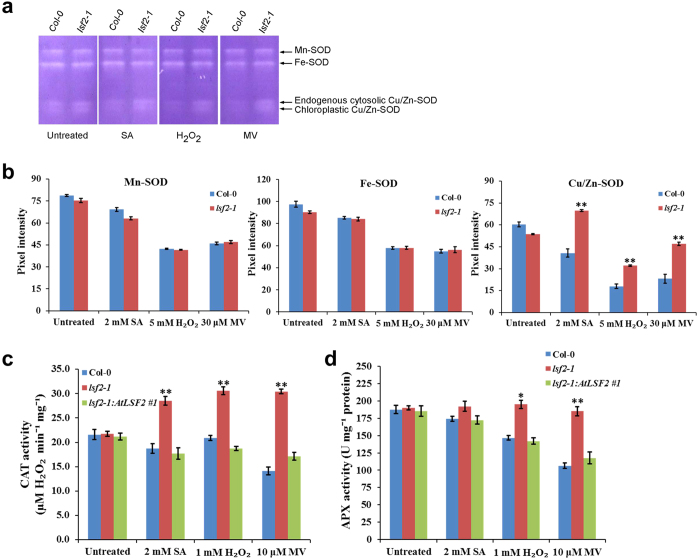
The activities of ROS antioxidant enzymes in plants. (**a**) SOD activity was examined by NBT staining after non-denaturing PAGE. (**b**) Pixel intensity of SODs was calculated using a plot profile. CAT activity (**c**) and APX activity (**d**) were examined after pre-treatment with SA, H_2_O_2_ or MV (n = 3; student’s t-test, *P < 0.05; **P < 0.01).

By determining the levels of ROS in the *lsf2-1* mutant and wild type Col-0 plants, we found that the species of O_2_^•−^ and H_2_O_2_ were altered in the *lsf2-1* mutant under external treatment of several oxidative stress inducers. Therefore, we planned to investigate the activities of several enzymes involved in ROS homeostasis such as SOD, APX and CAT in *lsf2-1* mutant under oxidative stress. In plants, SOD represents the first line of defense against excess ROS by catalyzing the dismutation of O_2_^•−^ to H_2_O_2_^23^. Plants contain four types of SODs: Fe-SOD is present in plastids, Mn-SOD is mainly located in the mitochondria, and Cu/Zn-SOD isoforms are normally present in the cytosol and chloroplasts. Here, we investigated SOD activities through NBT staining following non-denaturing polyacrylamide gel electrophoresis, and found that there were no obvious differences in the four SOD profiles (as an indicator of SOD activities) between *lsf2-1* mutant and Col-0 plants under normal growth conditions (Fig. 2a, untreated). When the seedlings were exposed to inducers of oxidative stress such as SA, H_2_O_2_, or MV, no change in Mn-SOD or Fe-SOD activity was observed in Col-0 and *lsf2-1* mutant plants; but the activities of endogenous cytosol Cu/Zn-SOD and chloroplast Cu/Zn-SOD were less inhibited in *lsf2-1* compared with Col-0 under oxidative stress conditions (Fig. 2a,b), suggesting that the activities of Cu/Zn-SODs were more strongly inhibited in the presence of *Arabidopsis* LSF2 under oxidative stress.

CAT activity is measured by monitoring the extinction of H_2_O_2_, whereas APX activity is measured by monitoring ascorbate oxidization. Since endogenous H_2_O_2_ levels increased much more strongly in *lsf2-1* mutant than in Col-0 plants under oxidative stress conditions, we analyzed the activities of CAT and APX in Col-0, *lsf2-1*, and *lsf2-1:AtLSF2 #*1 plants. Under normal growth conditions, no obvious differences in the activities of these enzymes were detected in *lsf2-1* mutant compared with Col-0 plants (Fig. 2c,d, untreated). When the seedlings were treated with SA, H_2_O_2_, or MV, reduced CAT and APX activity were observed in Col-0, but more significant increases in CAT activity were observed in *lsf2-1* mutant (Fig. 2c). However, APX activity was not altered in *lsf2-1* in response to the various oxidative stress conditions (Fig. 2d). When seedlings were under oxidative stress, CAT and APX activity were inhibited in the presence of LSF2, whereas CAT activity increased in the absence of LSF2, and APX activity was not altered in the absence of this protein. Therefore, CAT, not APX, is mainly responsible for H_2_O_2_ scavenging in the absence of *Arabidopsis* LSF2 under oxidative stress conditions.

### Root development is affected by oxidative stresses in *lsf2-1* mutant

**Figure 3:**
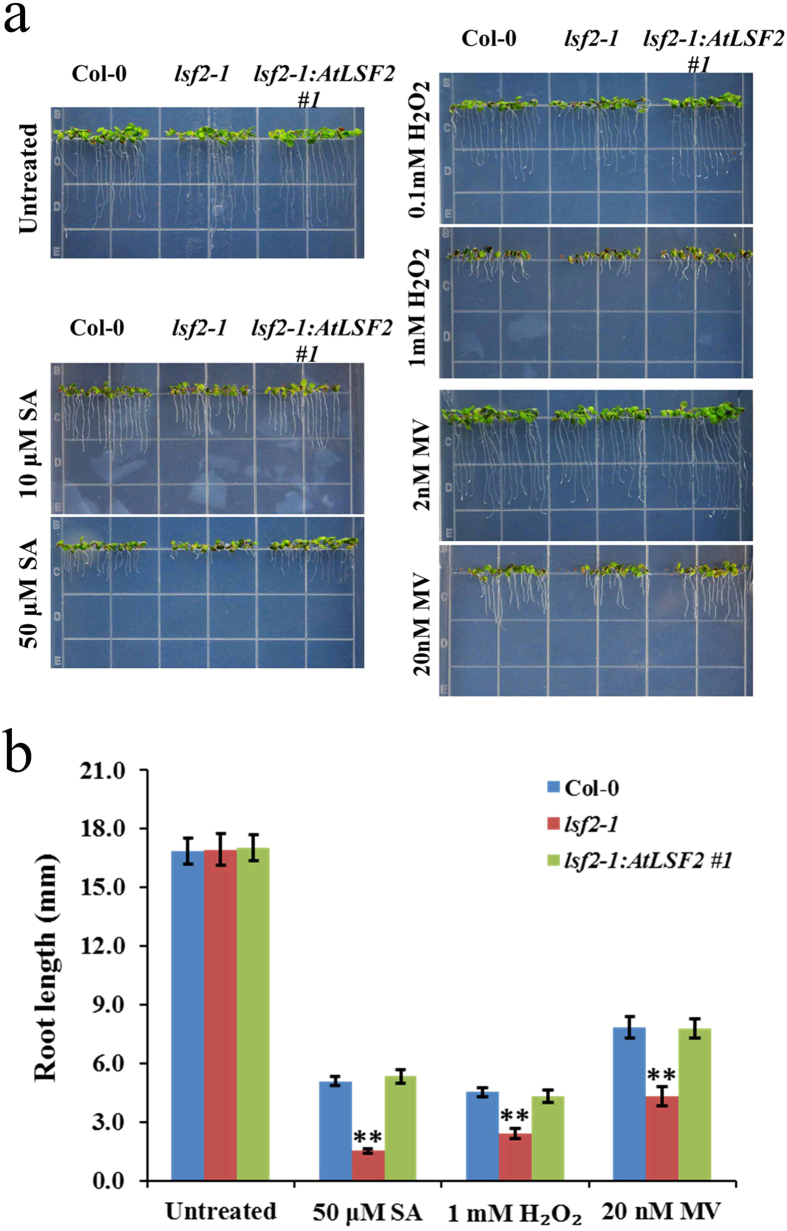
Phenotypic analysis of root growth in *lsf2-1* mutant and wild type plants under oxidative stress conditions. (**a**) Seedlings were grown under oxidative stress. (**b**) The assay of root length of seedlings, which were grown on MS medium containing 50 μM SA, 1 mM H_2_O_2_, or 20 nM MV (n = 20; student’s t-test, **P < 0.01).

Based on the altered activities of ROS scavenging enzymes such as SOD, APX and CAT in *lsf2-1* and wild type Col-0 plants under several oxidative stress conditions, it seems that LSF2 might be involved in differential regulation of these above mentioned enzymes, which could be the reason for the altered levels of ROS. Previous studies have suggested that ROS level also regulates the root length and root hair development^1^,^24^. Therefore, we have performed the detail phenotypic assays to investigate the root growth and development under various oxidative stress conditions. Under normal growth conditions, the phenotype of *lsf2-1* mutant was not significantly different from that of Col-0. However, when treated with exogenous oxidative stress via exposure to SA, H_2_O_2_, or MV, *lsf2-1* mutant plants grew poorly compared with Col-0 plants (Fig. 3a,b). Specifically, *lsf2-1* roots were shorter than Col-0 roots on medium containing 10 μM SA, 0.1 M H_2_O_2_, or 2 nM MV (Fig. 3a). The differences in root length between Col-0 and *lsf2-1* became more pronounced when grown on medium containing higher concentrations of these agents, such as 50 μM SA, 1 mM H_2_O_2_, or 20 nM MV (Fig. 3a,b). These findings indicate that the root growth of *lsf2-1* is more strongly inhibited than that of Col-0 under oxidative stress conditions, suggesting that *lsf2-1* mutant is hypersensitive to oxidative stress.

**Figure 4:**
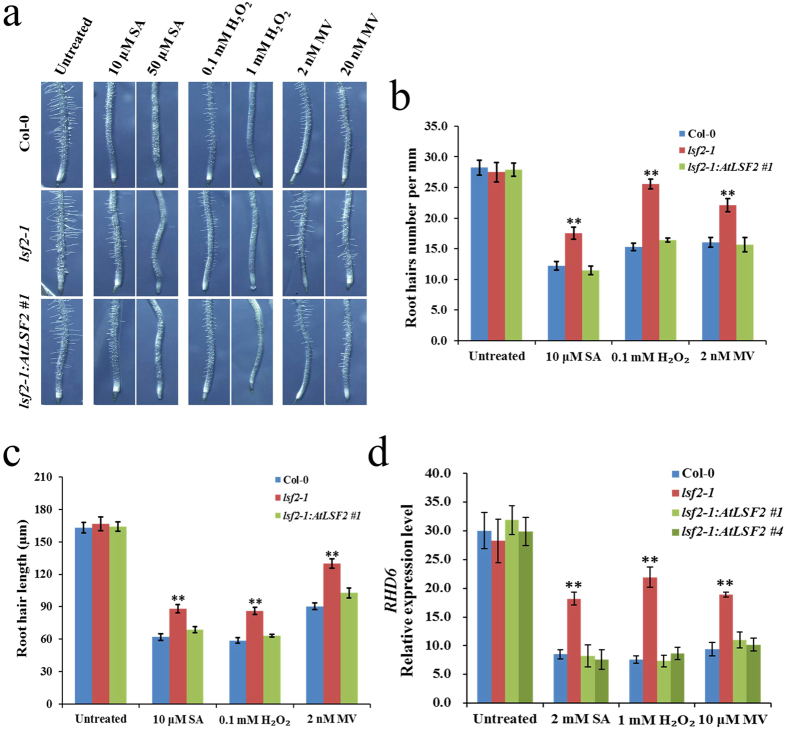
Phenotypic analysis of root hair development in *lsf2-1* mutant and wild type plants under oxidative stress conditions. Morphology of root hairs (**a**) of seedlings was grown under oxidative stress. Average number of root hairs per mm (**b**) and root hair length (**c**) were determined in seedlings which were grown on MS medium containing 10 μM SA, 0.1 mM H_2_O_2_, or 2 nM MV (n = 20; student’s t-test, **P < 0.01). (**d**) The relative expression of *RHD6* under oxidative stress was detected by qPCR (n = 3; student’s t-test, **P < 0.01).

No significant differences were found in root hair length or root hair number between *lsf2-1* mutant and Col-0 plants under normal growth conditions (Fig. 4a, untreated). However, when treated with oxidative stress via exposure to high concentrations of oxidative stress-inducing agents (50 μM SA, 1 mM H_2_O_2_, or 20 nM MV), root hair formation was almost completely inhibited in both Col-0 and *lsf2-1* mutant (Fig. 4a). When grown on lower concentrations of these agents (such as 10 μM SA, 0.1 mM H_2_O_2_, or 2 nM MV), the root hair density of *lsf2-1* was higher than those of Col-0 (Fig. 4b), and the root hairs length of *lsf2-1* were much longer than those of Col-0 (Fig. 4c). We also examined the expression of *ROOT HAIR DEFECTIVE6* (*RHD6*), which positively regulates root hair development^25^. The transcriptional expression of *RHD6* was very significantly higher in *lsf2-1* than in Col-0 under oxidative stress conditions (Fig. 4d). Together, these results indicate that deficiency of LSF2 in *Arabidopsis* results in less inhibition of root hair development, as well as less inhibition of *RHD6* expression, under oxidative stress conditions compared with normal growth condition (Fig. 4d, untreated).

### *Arabidopsis* LSF2 interacts with MPK8 *in vivo*

**Figure 5:**
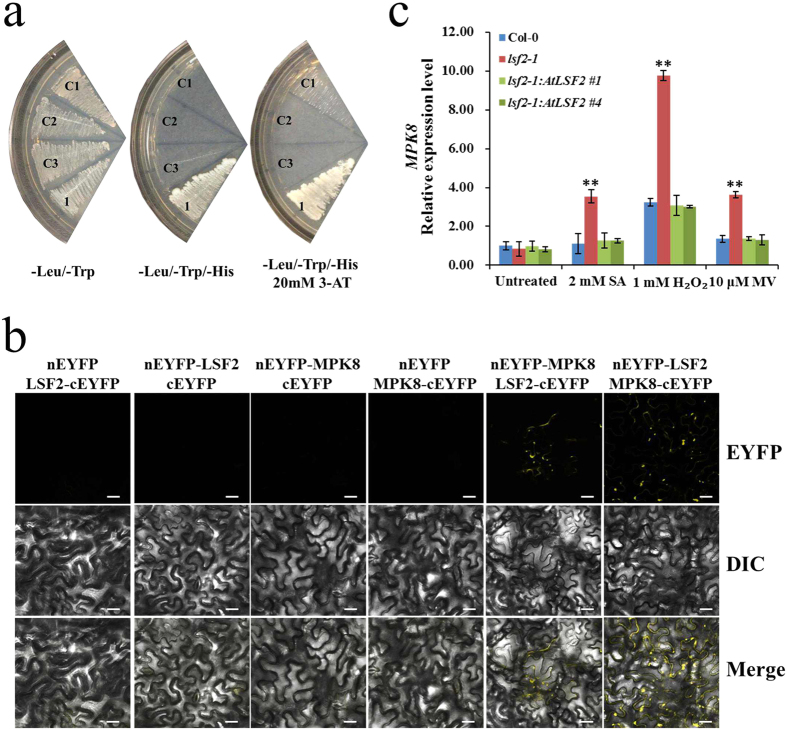
Interaction analysis of LSF2 and MPK8 by Y2H and BiFC methodologies. (**a**) Interaction between LSF2 and MPK8 was assayed by Y2H, C1: *AD* + *BD*; C2: *AD-LSF2* + *BD*; C3: *AD* + *BD-MPK8*; 1: *AD-LSF2* + *BD-MPK8*. (**b**) Fluorescence was observed due to complementation of fusion protein LSF2-cEYFP with nEYFP-MPK8, or nEYFP-LSF2 with MPK8-cEYFP, Bars = 20 μm. (**c**) The relative expression of *MPK8* under oxidative stress was detected by qPCR (n = 3; student’s t-test, **P < 0.01).

Previously, several MAP kinases have been shown to be involved in both biotic and abiotic stresses and affect the ROS homeostasis^26^. Also dual specificity phosphatases (DSPs) are known to regulate and target several MAP kinases in different abiotic stress condition, such as oxidative stress. To determine if LSF2, a dual specificity protein phosphatase, is also targeting any MAP kinase, we have performed a comprehensive yeast two-hybrid analysis with several MAP kinases (data not shown). Using yeast two-hybrid assays, we found that LSF2 interacted with MPK8 (AT1G18150) (Fig. 5a). To confirm this finding, we purified both full-length LSF2 fused with His_6_ tag and full-length MPK8 fused with glutathione-S-transferase (GST) from *E. coli* extracts, and then examined the possibility of LSF2 and MPK8 to interact *in vitro* via a pull down assay and western blotting with anti-GST antibody. The results showed that MPK8 interacted with LSF2 *in vitro* (Supplementary Fig. S3). To further examine the interaction between LSF2 and MPK8 *in vivo*, we performed a bimolecular fluorescence complementation (BiFC) assay. *Agrobacterium tumefacien* strain EHA105 containing expression binary vectors for *nEYFP-LSF2* together with *MPK8-cEYFP*, or *LSF2-cEYFP* together with *nEYFP-MPK8*, were co-infiltrated into *N. benthamiana* leaves, respectively. Coexpression of *nEYFP-LSF2* and *MPK8-cEYFP* (or *LSF2-cEYFP* and *nEYFP-MPK8*) resulted in strong fluorescent signals in the cytoplasm due to reconstitution of the fluorescent marker, EYFP (Fig. 5b). No fluorescent signals were observed when *nEYFP-LSF2* or *LSF2-cEYFP* was coexpressed with *cEYFP* or *nEYFP* empty vector, which served as negative controls. Interestingly, the transcriptional expression level of *MPK8*, not *respiratory burst oxidase homolog D* (*RbohD*), was enhanced under these oxidative stress conditions in leaves of *lsf2-1* mutant (Fig. 5c; Supplementary Fig. S4).

## Discussion

Protein phosphatases (PPs) play important roles in plants^15^. To date, only a few PPs have been characterized in *Arabidopsis* and other flowering plants. AtRP1 (PPDK regulatory protein) not only exhibits functional properties of protein phosphatases, but it also displays functional properties of protein kinases, as it regulates the activity of pyruvate orthophosphate dikinase (PPDK) via phosphorylation or dephosphorylation of its threonine residue^27^. Interestingly, the plastid-localized enzyme NADP-THIOREDOXIN REDUCTASE C (NTRC) regulates starch synthesis in chloroplasts and amyloplasts in response to either light or sucrose, and it is also involved in maintaining redox homeostasis in plastids of both photosynthetic and nonphotosynthetic tissues, thereby affecting the formation of lateral roots in *Arabidopsis*^28^,^29^. LSF2 is a dual specificity protein phosphatase that displays high activity toward *para*-nitrophenyl phosphate (pNPP) (Supplementary Fig. S5), and it dephosphorylates glucan to provide access for amylases that release maltose and glucose from starch in *Arabidopsis*^30^. When the effect of LSF2 null-mutation compared at morphological level, a severity of curly dwarf symptom in *lsf2-1* leaves was increased under SA treatment (Supplementary Fig. S6). This suggests the possibility that LSF2 could improve the tolerance to oxidative stresses in plant, which was correlated with the up-regulated expression of LSF2 in wild type leaves under oxidative stress conditions. Hence, the function of LSF2 is not only projected in the root development but LSF2 could also be involved in improving plant tolerance to oxidative stresses. The present results suggest that LSF2 also functions in maintaining cellular ROS homeostasis in plants.

Many environmental and genetic factors influence root growth and root hair formation, such as light, calcium, free radicals, and ethylene^31^. Changes in redox are involved in controlling plant growth and development, and ROS production is associated with root development^32^. NADPH oxidases and peroxidase have often been shown to be responsible for the production of endogenous O_2_^•−^ and H_2_O_2_ in plants. When H_2_O_2_ levels are reduced in plant, increased production of O_2_^•−^ is an important mechanism that maintains ROS homeostasis^11^. Redox regulation of AGPase is severely affected in leaves and roots of the *Arabidopsis ntrc* mutant, and introducing fully functional chloroplasts into this mutant by complementation is both necessary and sufficient for sustaining root growth and lateral root formation, with similar rates to those of the wild type^28^. The induction of *REDOX RESPONSIVE TRANSCRIPTION FACTOR1* (*RRTF1*) in plants results in ROS accumulation via ROS-generating biotic signals^33^. Therefore, the balance between O_2_^•−^ and H_2_O_2_ to maintain ROS homeostasis might be involved in root development in plants. Accordingly, in the present study, we investigated the production of O_2_^•−^ and H_2_O_2_ and the activities of several antioxidant enzymes in response to oxidative stresses, and we examined the associated effect of the absence of functional LSF2 on root development in *Arabidopsis*. Under oxidative stress conditions, the generation rate of O_2_^•−^ was obviously lower, and the level of endogenous H_2_O_2_ was higher, in the *lsf2-1* mutant. These changes are consistent with the changes in Cu/Zn-SODs, CAT, and APX activity observed in the *lsf2-1* mutant, which led to more inhibition of root growth and less inhibition of root hair development under oxidative stress conditions compared to Col-0 plants. Meanwhile, the expression of some of the marker genes involved in the regulation of root elongation, such as *SCN1*, *CPC* and *PIN3*, were analyzed between Col-0 and *lsf2-1* mutant, but no significant difference was found (Supplementary Fig. S7). Furthermore, the expressions of marker genes involved in the regulation of root hairs also were determined by qPCR, including *RHD6* gene. RHD6 regulates the development of root hair-forming cells, and *Arabidopsis rhd6* mutants have fewer root hairs than the wild type^25^. A recent report showed that ROS production is induced by vanadate treatment and that RHD6 is involved in vanadate-induced root hair initiation^34^. In the present study, the lack of functional LSF2 resulted in less reduced expression of *RHD6* under oxidative stress in *Arabidopsis*, which is consistent with the reduced inhibition of root hair development in the *lsf2-1* mutant compared with Col-0 plants. Collectively, the results demonstrate that LSF2 affects root development through modulating ROS homeostasis in *Arabidopsis*.

Maintaining steady ROS levels appears to require a network involving at least 152 genes in *Arabidopsis*^35^. PFT1 mainly regulates ROS-related gene expression to control the formation of root hairs via ROS homeostasis. Lower levels of H_2_O_2_ are correlated with the reduced expression of *POX*, and higher levels of O_2_^•−^ are correlated with the increased expression of *NADPH*^13^. A recent study demonstrated that *Mitogen-activated protein kinase kinase kinase 1* (*MEKK1*) is involved in cellular redox control and integrating ROS homeostasis with plant development and hormone signaling, and MPK4 is the downstream target of MEKK1 in plants^36^,^37^. Ca^2+^ influx and Ca^2+^/Calmodulins (CaMs) regulate ROS homeostasis and the activities of all antioxidant enzymes. MPK6 mediates Ca^2+^ influx across the plasma membranes of root cells to control H_2_O_2_-induced root elongation in *Arabidopsis*^38^. *Arabidopsis* MPK8 interacts with CaM3, CaM4, and CaM7 in the cytoplasm, and is activated by CaMs and mitogen-activated protein kinase kinase 3 (MKK3) to help maintain ROS homeostasis^39^. After mechanical wounding, ROS accumulation increases in the wounding sites of the *mpk8* mutant, whereas ROS accumulation decreases in the tissues of *MPK8-GFP* plants. Furthermore, MPK8 has a conserved phosphorylation motif-TDY and is dual phosphorylated in *Arabidopsis* pollen at Thr and Tyr residues in the TDY motif^40^. LSF2, a dual specificity protein phosphatase, dephosphorylates phospho-Ser, -Thr, and -Tyr in proteinaceous substrates. We propose that LSF2 might also inactivate MPK8 in *Arabidopsis* through dephosphorylation. Our results also show that production of ROS in *lsf2-1* mutant is independent of RbohD, which functions as an essential ROS regulator. However, the expression of *MPK8* is induced under these oxidative stress conditions in leaves of *lsf2-1* mutant, and LSF2 interacts with MPK8 in the cytoplasm, suggesting that LSF2 regulates ROS homeostasis through interaction with MPK8 in response to oxidative stress in *Arabidopsis*.

## Materials and Methods

### Plant materials

All plant materials used in this study were in the ecotype Columbia-0 (Col-0) *Arabidopsis thaliana* background. The *lsf2-1* allele is the SAIL line 595_F04 of *AT3G10940* and was obtained from the *Arabidopsis* Biological Resource Center (ABRC). To produce the construct used for functional complementation of the *lsf2-1* mutant, the genomic DNA fragment of *LSF2* (*gLSF2*) including the 5′promoter region, coding region, 3′UTR, and following 1282 bp sequence was amplified with the primer pair *gLSF2_*Fw and *gLSF2_*Rv (Supplementary Table S1). The resulting fragment was cloned into entry vector *pENTR-TOPO* (Invitrogen), followed by cloning into binary vector *pBA002a*. The construct *pBA002a-gLSF2* was transformed into the EHA105 strain of *Agrobacterium tumefaciens*, followed by transformation into *lsf2-1* mutant plants by the floral dip method^41^. Transgenic plants harboring *gLSF2* were selected on MS medium plates containing 50 μg/ml BASTA. T_1_ seedlings expressing g*LSF2* were grown in soil, and homozygous plants of the four independent complementary lines (*lsf2-1:AtLSF2*) were identified after three rounds of BASTA application. The insertion site of *gLSF2* was determined by genome walking kit (Takara) with three specific primers (SP1, SP2 and SP3; Supplementary Table S1).

*Arabidopsis* seeds were surface-sterilized, and imbibed for 2 days at 4 °C in darkness for stratification, then germinated on MS medium solidified with 0.8% agar in Petri plates. Plants were grown in growth chamber under controlled conditions, 22 °C day/18 °C night with a relative humidity of 60% under long-day conditions (16-h light illumination of 150 μmol·m^−2^·sec^−1^, and 8-h dark cycle).

### RNA extraction and qPCR analysis

Total RNA was extracted from *Arabidopsis* using an RNeasy Plant Mini kit (Qiagen). Reverse transcription was performed with 2 μg each of total RNA and oligo (dT)_18_ primers using the SuperScript II RT kit (Invitrogen). The cDNA was mixed with SYBR premix Ex Taq (TaKaRa) and gene-specific primers, and amplified in a Bio-Rad CFX96 real-time system. Expression of *ACTIN2* was used as a control for cDNA levels. The primer sequences used for qPCR are listed in Supplementary Table S1.

### Determination of O_2_^•−^ generation and endogenous H_2_O_2_ contents

Pre-treating 2-week-old Col-0, *lsf2-1*, and *lsf2-1:AtLSF2* #1 plants with 2 mM SA, 1 mM H_2_O_2_, or 10 μM MV for 30 min, O_2_^•−^ generation of seedlings was measured with the XTT assay, as described by Liszkay^2^, and endogenous H_2_O_2_ contents were quantified using a peroxidase enzyme assay according to the method of Youn-Il Park^42^. In addition, nitro blue tetrazolium (NBT) staining also was performed as described previously^43^. When NBT reacts with O_2_^•−^, the color shifts from pale yellow to dark blue, which is used to monitor the generation of superoxide radicals in a histo-chemical staining procedure. The roots were observed under a stereoscopic microscope (Olympus SZX16, Olympus Japan Co., Tokyo, Japan).

### Antioxidant enzyme activity assays: superoxide dismutases (SODs), catalase (CAT), and ascorbate peroxidase (APX)

Pre-treating 2-week-old Col-0 and *lsf2-1* plants with exogenous 2 mM SA for 3 h, 5 mM H_2_O_2_ for 30 min, or 30 μM MV for 2 h, SOD activity was detected using NBT staining following electrophoresis as described^44^. Separation of SOD isoforms was performed with each 100 μg protein sample by non-denaturing polyacrylamide gel electrophoresis (PAGE) at 4 °C using a 12% resolving gel and a 5% stacking gel. While, pre-treating 2-week-old Col-0, *lsf2-1*, and *lsf2-1:AtLSF2* #1 plants with exogenous 2 mM SA, 1 mM H_2_O_2_, or 10 μM MV for 30 min, CAT activity was determined based on the decrease in absorbance at 240 nm through the extinction of H_2_O_2_^45^, and APX activity was determined based on the decrease in absorbance at 290 nm due to ascorbate oxidation^46^.

### Root growth and root hair development assays

Col-0, *lsf2-1*, and *lsf2-1:AtLSF2* #1 seeds were surface sterilized and germinated in Petri plates containing MS medium. Exogenous oxidative treatments were performed by adding different concentrations of SA, H_2_O_2_, or MV (as noted in the figure) to the MS medium. Four- to five-day-old seedlings were transferred individually to defined MS medium. The initial position of the seedling root apex was marked, and the increase in root length was measured after an 8-day incubation. Root growth was also photo-recorded with a digital camera (Nikon D90). The length and density of root hairs were assayed under a stereoscopic microscope (Olympus SZX16). Root growth was calculated with Image J software based on the differences from photographs of the respective time points and plates.

### Yeast two-hybrid (Y2H) assay

The Matchmaker GAL4-based two-hybrid system (Clontech) was used to perform yeast two-hybrid assays. The cDNAs encoding full-length LSF2 were cloned into the *pGAD424* vector (Clontech) to generate activation domain (AD) constructs. The cDNA encoding full-length MPK8 was cloned into the *pGBT9* vector (Clontech) to generate binding domain (BD) constructs. All constructs and empty vector controls were transformed into yeast stain AH109 using the modified lithium acetate method. Co-transformed yeast transformants were selected on plates containing SD/-Leu/-Trp medium and screened on selective medium containing SD/-Leu/-Trp/-His with 20 mM 3-amino-1,2,4,-triazole (3-AT) to test protein interactions, and empty vectors *pGBT9* and *pGAD424* were used as negative controls.

### Expression of recombinant proteins and pull-down assay

Full-length *LSF2* cDNA was cloned into *pET-28a* (+) (Novagen) to generate the protein expression vector for biosynthesis of the fusion protein LSF2-His_6_. The cDNA encoding full-length MPK8 was inserted into *pGEX* to generate the translational fusion of GST-MPK8. All recombinant vectors were transformed into *E. coli* BL21 cells. Isopropyl-β-D-thiogalactoside (IPTG) was used to induce the expression of fusion proteins. GST fusion proteins were purified on glutathione Sepharose^TM^ R10-Flammable (GE Health), and His_6_ fusion protein was purified on Ni^2+^-nitrilotriacetate (Ni^2+^ -NTA) resins (Qiagen). Prey protein LSF2-His_6_ and bait fusion protein GST-MPK8 were then added to 1 ml of binding buffer (50 mM Tris-HCl pH 7.5, 100 mM NaCl, 0.5% Triton X-100, 0.5 mM β-mercaptoethanol, and 2% proteinase inhibiter cocktail) and incubated with amylose resin beads at 25 °C for 1 h. After incubation, the beads were washed six times with fresh binding buffer. Pull-down proteins were separated by SDS-polyacrylamide gel electrophoresis at on 7.5% resolving gel and detected by western blotting using anti-GST antibody (Santa Cruz Biotechnology).

### Bimolecular Fluorescence Complementation (BiFC) assay

The vectors *pBA-cEYFP*(*175-end*)*-N1* (*pBA3032*) and *pBA-nEYFP (1-174)-C1* (*pBA3036*) were used as BiFC Gateway vectors, which respectively generate *cDNA-cEYFP* and *nEYFP-cDNA*^47^. The cDNA encoding full-length LSF2 or MPK8 was cloned into the BiFC Gateway vectors, respectively, to test interactions of the proteins *in vivo*. Recombinant plasmids encoding cEYFP and nEYFP fusions were transformed into competent *Agrobacterium tumefaciens* (strain EHA105) cells. The EHA105 transformants with recombinant plasmid were suspended in 10 mM MgCl_2_ and 150 μM acetosyringone. The mixtures were incubated at 25 °C for at least 3 h without shaking and co-infiltrated into *Nicotiana benthamiana* leaves. Microscopic observations of infiltrated plants were performed after 2–3 days. The unfused protein cEYFP or nEYFP was used as a negative control.

## Additional Information

**How to cite this article**: Zhao, P. *et al*. The *LIKE SEX FOUR2* regulates root development by modulating reactive oxygen species homeostasis in *Arabidopsis*. *Sci. Rep.* **6**, 28683; doi: 10.1038/srep28683 (2016).

## Supplementary Material

Supplementary Information
