# Supplementary material for: The LIKE SEX FOUR2 regulates root development by modulating reactive oxygen species homeostasis in Arabidopsis
Source: Sci Rep. 2016 Jun 28;6:28683. doi: 10.1038/srep28683 (PMC4923905; doi:10.1038/srep28683)
Supplement: Supplementary Information [file srep28683-s1.pdf]

The *LIKE SEX FOUR2* regulates root development by modulating reactive oxygen species homeostasis in *Arabidopsis*

Pingzhi Zhao[1※], Lubomir N. Sokolov [1※], Jian Ye[3], Cheng-Yi Tang[1], Jisen Shi[4], Yan Zhen[4], Wenzhi Lan[1], Zhi Hong[1], Jinliang Qi[1], Gui-Hua Lu[1*], Girdhar K. Pandey[2*] & Yong-Hua Yang[1*]

Supplementary information

Figure S1. Molecular characterization of the *lsf2-1* mutant line.

a. Schematic representation of the *lsf2-1* allele, with the T-DNA insertion shown as inverted triangles.

b. Genotyping of the *lsf2-1* mutant. P1, P2, and SAIL_LB3 represent the specific primers used to screen and confirm the homozygous line of the *lsf2-1* mutant by RT-PCR. Genomic DNA from Col-0 plants was used as a positive control.

c. Analysis of *LSF2* expression by RT-PCR in Col-0 and *lsf2-1* mutant. Transcriptional expression of *ACTIN2* was used as a positive control.

d. Relative expression levels of *LSF2* in leave and root of Col-0 plants under oxidative stress conditions. Data represent mean values ± SE (n =6). The mean value of *LSF2* expression in leaves was significantly different from that of root as determined by student’s t-test analysis (**, P < 0.01).

Figure S2. Molecular characterization of functional complementation of the *lsf2-1* mutant (*lsf2-1:AtLSF2*).

a. Genomic insertion location of *gLSF2* in *lsf2-1:AtLSF2* transgenic lines. The schematic diagram shows *Arabidopsis* genes. The *gLSF2* insertion position is indicated by an arrow. White box, UTR; Gray box, exon coding region; black line, intron region; dotted line, intergenic region.

b. Relative expression levels of *LSF2* in Col-0, *lsf2-1,* and four complemented lines of *lsf2-1:AtLSF2* were determined. Data represent mean values ± SE (n =6).

Figure S3. *In vitro* pull-down assays investigating the interaction between LSF2 and MPK8.

LSF2-His_6 was used to pull down GST or GST-MPK8. Immunoblot analysis was performed using anti-GST antibody to detect the associated proteins.

Figure S4. The expression level of *RbohD* in *lsf2-1* mutant and wild type plants was detected under oxidative stress conditions.

The relative expression of *RbohD* under oxidative stress (SA, H_2O_2 or MV) was detected by qPCR in Col-0 and *lsf2-1* plants. Data represent mean values ±SE (n =6). *ACTIN2* was used as a positive control.

Figure S5. LSF2 phosphatase activity assay.

Time course of protein phosphatase activity of LSF2 was examined, with *para*-nitrophenyl phosphate (pNPP) used as substrate.

Figure S6. Phenotypic analysis of wild type, *lsf2-1* mutant and complemented line under SA induced oxidative stress.

Under 50 μM SA treatment, the severity of curly dwarf symptom in *lsf2-1* leaves was increased as compared to Col-0 and complemented line (*lsf2-1:AtLSF2* #1) plants.

Figure S7. The expression levels of *SCN1*, *CPC* and *PIN3* in *lsf2-1* mutant and wild type plants were detected under oxidative stress conditions.

The relative expression of *SCN1* (**a**), *CPC* (**b**) and *PIN3* (**c**) under oxidative stress (SA, H_2O_2 or MV) was detected by qPCR in Col-0 and *lsf2-1* plants. Data represent mean values ±SE (n =6). *ACTIN2* was used as a positive control.

Table S1. Primer sequences used in this study.

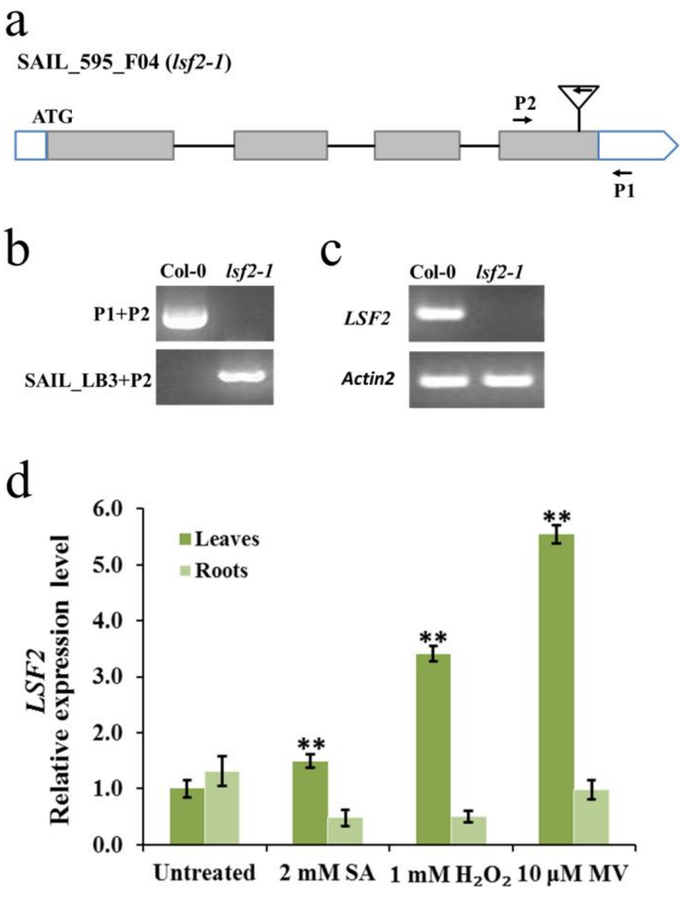

Figure S1. Molecular characterization of the *lsf2-1* mutant line.

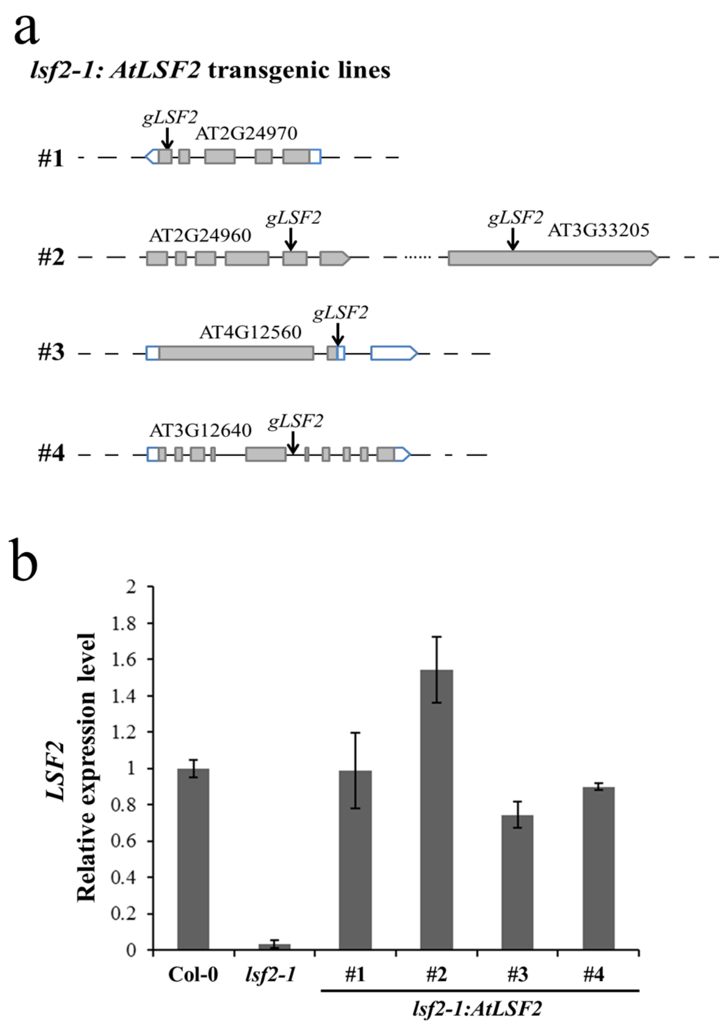

Figure S2. Molecular characterization of functional complementation of the *lsf2-1* mutant (*lsf2-1:AtLSF2*).

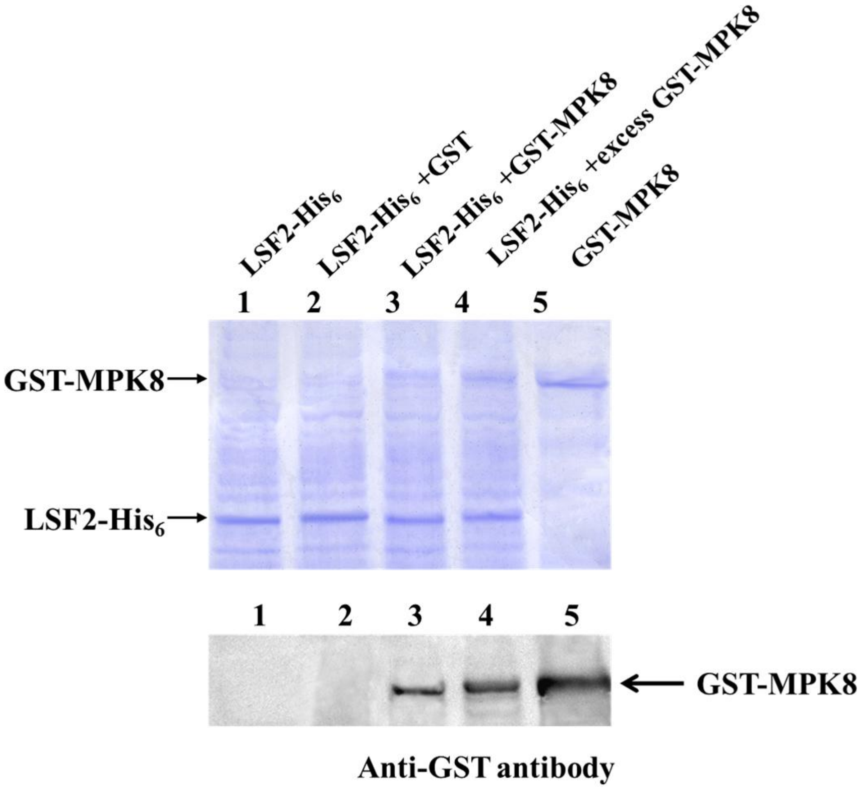

Figure S3. ***In vitro*** **pull-down assays investigating the interaction between LSF2 and MPK8**

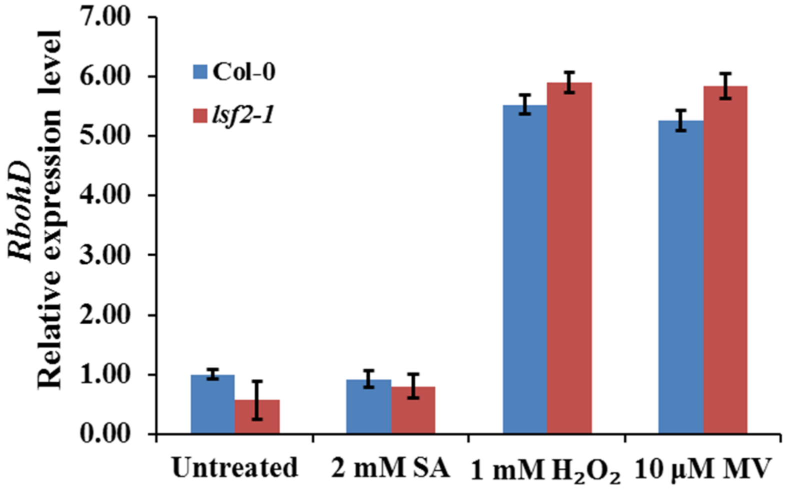

Figure S4. The expression level of ***RbohD*** **in** ***lsf2-1*** **mutant and wild type plants was detected under oxidative stress conditions.**

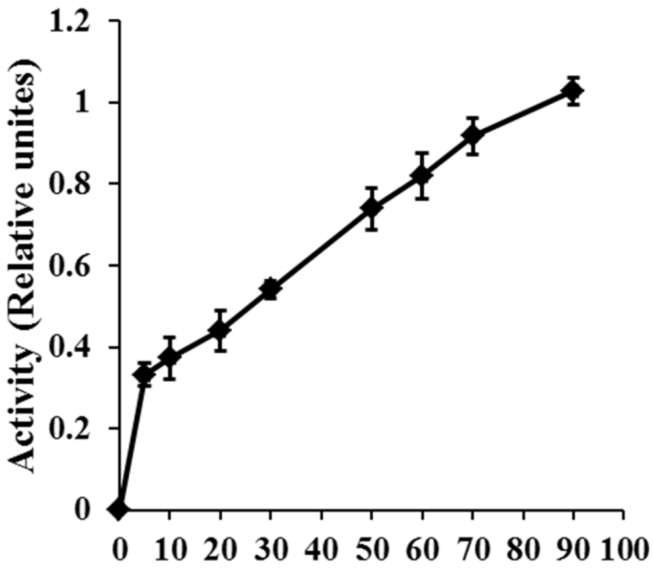

Figure S5. LSF2 phosphatase activity assay

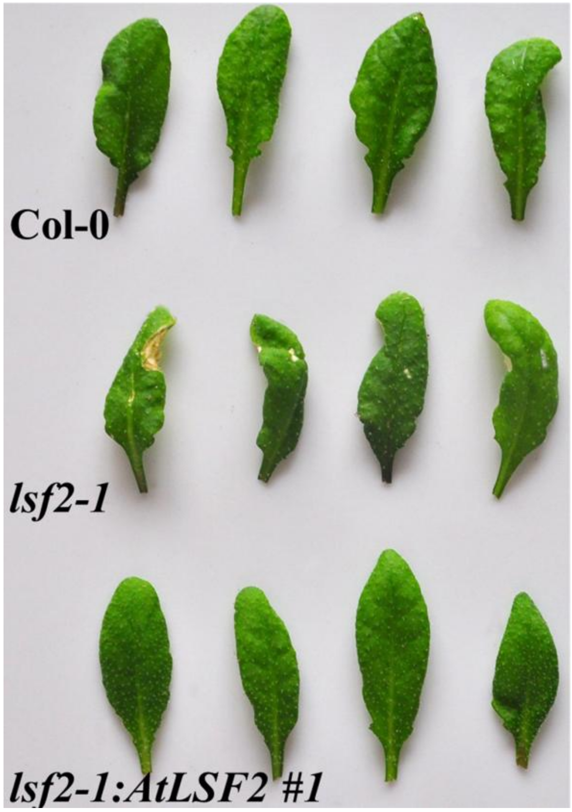

Figure S6. Phenotypic analysis of wild type, *lsf2-1* mutant and complemented line under SA induced oxidative stress.

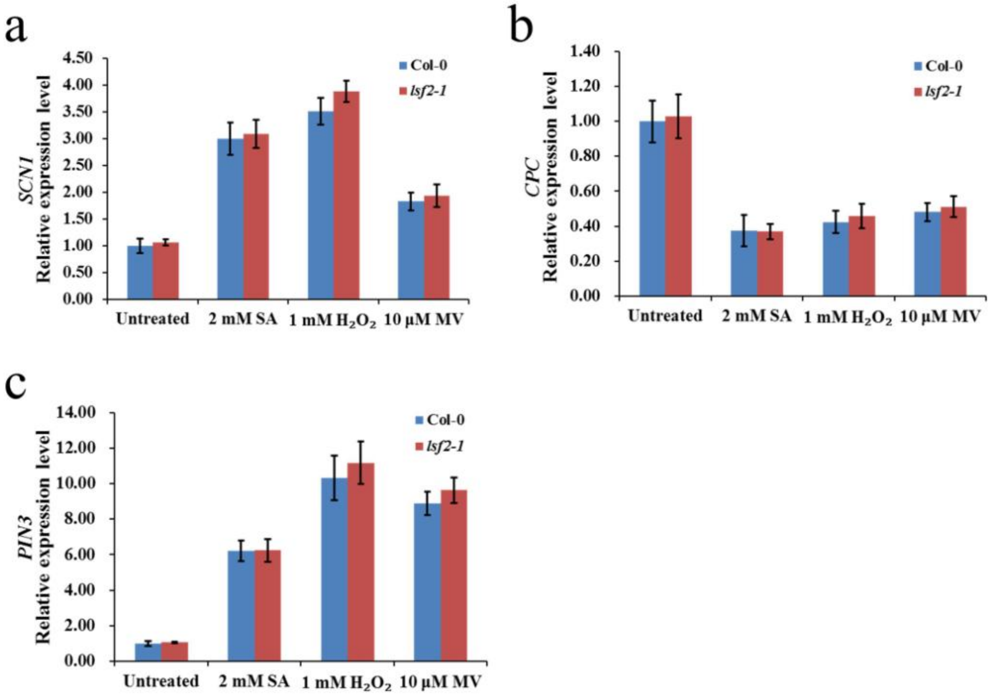

Figure S7. The expression levels of *SCN1*, *CPC* and *PIN3* in *lsf2-1* mutant and wild type plants were detected under oxidative stress conditions.

Table S1. Primer sequences used in this study.

SAIL_LB3		TAGCATCTGAATTTCATAACCAATCTC
P1		GGTATCAAAGCGTCCATGTGG
P2		GTCGTGCCATCACTTTAACAGT
LSF2	Fw	ATGAGTGTGATTGGAAGCAAG
	Rv	TCAGGTTCCACGGAGGGCCC
ACTIN2	Fw	CTGTTCTCTCCTTGTACGCCAGT
	Rv	CGGGTAATTCATAGTTCTTCTCGAT
gLSF2	Fw	TTTCGCC GGCTGAGGGAGAAAAGCAGACAATGTGC
	Rv	GATTAGTCTTGCTTGGTTTGTAAAATGATGAAG
LSF2-qPCR	Fw	AGATTTTGATCCACTTTCGTTG
	Rv	GTTCTTGAATCAACTTCCTTTC
RHD6-qPCR	Fw	GCCCTAGATCCACCGAAACTCC
	Rv	TGGCTGCTAGGCTTTGTGG
MPK8	Fw	CAAGGGTACCATGGGTGGTGGTGGGAATCTCG
	Rv	CAAGCTCGAGGAATTGTGAAGAGAAGCA
MPK8-qPCR	Fw	CCTTCTGGAACGCCTGCTTG
	Rv	GCTGATCACCACCACGAAGG
Specific primers for screening independent complementary transgenic lines	SP1	TGATATCTAGGGACCTGCAGGCATGC
	SP2	AAAGCCAGTCCGCAGAAACGGT
	SP3	CTTGCAGTGGGCTTACATGGCG
SCN1-qPCR	Fw	ACTCTTGATCCGGAAGTGAGG
	Rv	TGTCCACTTTGACACCGG
CPC-qPCR	Fw	TGGATAAACGACGACGG
	Rv	ACGCCGTGTTTCATAAGC
PIN3-qPCR	Fw	TTAGTTTGGGGTTGTTCATGG
	Rv	CCCGAGTAGAATGTAGTAAACC
